# CircHIPK3 Plays Vital Roles in Cardiovascular Disease

**DOI:** 10.3389/fcvm.2021.733248

**Published:** 2021-09-29

**Authors:** Lei Zhang, Yin Wang, Fei Yu, Xin Li, Huijuan Gao, Peifeng Li

**Affiliations:** Institute for Translational Medicine, The Affiliated Hospital of Qingdao University, Qingdao University, Qingdao, China

**Keywords:** circular RNAs, circHIPK3, cardiovascular disease, pathogenesis, underlying mechanisms

## Abstract

Circular RNAs (circRNAs) are covalently closed RNAs that function in various physiological and pathological processes. CircRNAs are widely involved in the development of cardiovascular disease (CVD), one of the leading causes of morbidity and mortality worldwide. CircHIPK3 is generated from the second exon of the *HIPK3* gene, a corepressor of homeodomain transcription factors. As an exonic circRNA (ecRNA), circHIPK3 is produced through intron-pairing driven circularization facilitated by Alu elements. In the past 5 years, a growing number of studies have revealed the multifunctional roles of circHIPK3 in different diseases, such as cancer and CVD. CircHIPK3 mainly participates in CVD pathogenesis through interacting with miRNAs. This paper summarizes the current literature on the biogenesis and functions of circHIPK3, elucidates the role of circHIPK3 in different CVD patterns, and explores future perspectives.

## Introduction

Non-coding RNAs are a group of RNAs that do not encode proteins, such as tRNA, rRNA, microRNA (miRNA), snRNAs, snoRNAs, long non-coding RNA (lncRNA), circRNA, etc. Several recent studies on miRNAs and lncRNAs illustrate their biological functions (1–6). CircRNAs were first discovered in 1976 in plant viruses (7), and were considered to have no function for a long time (8–10). However, with the rapid development of research methods and technologies, it has been proven that circRNAs have essential biological functions. They participate in the occurrence and development of many diseases, such as cancer (11–15) and CVD (16–20). There are several patterns of CVD, some of which could be fatal, such as myocardial infarction (MI), heart failure (HF) and coronary heart disease (CAD).

CircRNAs function through four different mechanisms: acting as miRNA or RNA binding protein (RBP) sponges (15, 21, 22), regulating the expression of their parental genes (23, 24), or acting as templates for protein translation (13, 25). CircHIPK3 is one of the most studied circRNAs in the past 5 years (26–30). CircHIPK3 was first determined to have biological functions in cancer studies (26, 31). Subsequently, its role in CVD was also established (32–34). In this review, we summarize the current knowledge on the biogenesis and underlying mechanisms of circHIPK3, and review the role of circHIPK3 in CVD for the first time.

## Biogenesis of circRNAs

CircRNAs are covalently circularized RNAs. CircRNAs are usually ~500 nt in length (35) and have high stability due to their covalently closed structures (15). CircRNAs are comprised of three different types: ecRNAs (36), exon-intron circRNAs (EIciRNAs) (24) and circular intronic RNAs (ciRNAs) (23). EcRNAs can be transported into cytosol (21, 37), whereas the other two types of circRNAs are confined to the nucleus due to their intron sequences (21, 23, 37).

**Figure 1:**
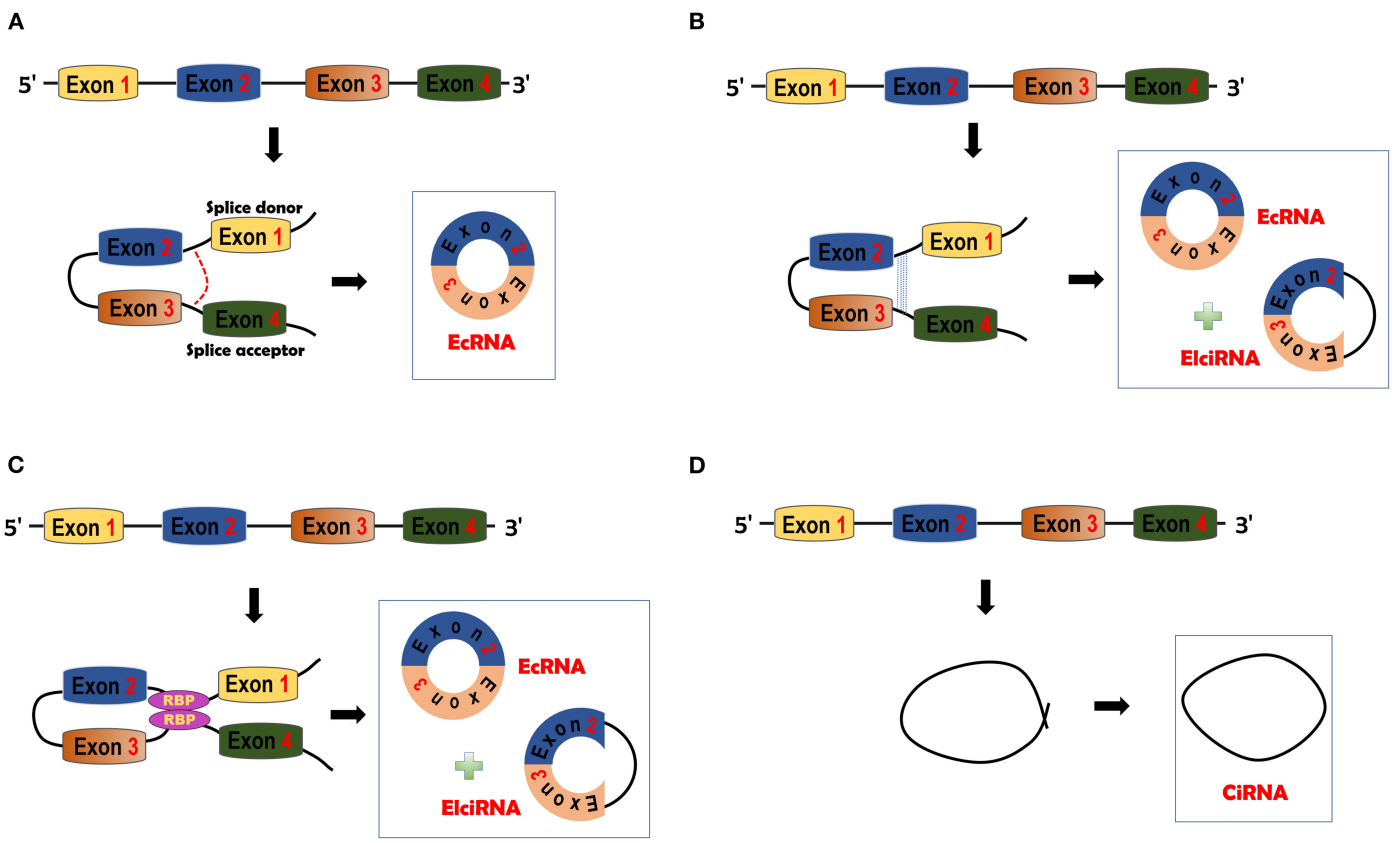
circRNA biogenesis. **(A)** Lariat-driven circularization model. The splice donor and acceptor can be bound to form exon-containing lariats. Further splicing will generate exonic circRNAs (ecRNAs). **(B)** Intron pairing-driven circularization model. This model is facilitated by the complementary pairing of RNA base motifs (e.g., Alu repeats) in introns. EcRNAs or Exon-intron circRNAs (EIciRNAs) can be formed by this model. **(C)** RNA binding protein (RBP)-driven model. RBPs can bind with each other and serve as a bridge of pre-mRNAs. Bridging can facilitate the formation of ecRNAs or EIciRNAs. **(D)** Circular intronic RNA (ciRNA) is formed by Fwith other sequences eliminated by spliceosomes.

CircRNAs can be generated through four different mechanisms. In the lariat-driven circularization model, only ecRNAs are produced. The GU motif in the 5′ end of introns (splice donor) and the AG motif (splice acceptor) in the 3′ end of introns can form a lariat. The lariat will be spliced by the splicesome and then ecRNAs will be made (38, 39) (Figure 1A). In the intron-pairing driven circularization model, either ecRNAs or EIciRNAs are generated. Intronic RNA base motifs, such as Alu repeats, can pair with the reverse complementary sequences to trigger direct cyclization. Circularization will cause the formation of EIciRNAs (introns retained) or ecRNAs (introns removed) (38) (Figure 1B). In RBP-mediated circularization, either ecRNAs or EIciRNAs are generated (22, 40). RBPs, such as muscleblind (MBL) proteins and Quaking (QKI) (22, 40), can dimerize to form a bridge that will pull two flanking introns close together, thereby stimulating backsplicing (Figure 1C). CiRNAs are formed from a different mechanism. GU-rich sequences close to the 5′ splice site of introns can bind with C-rich sequences close to the branch (23). The binding facilitates the formation of ciRNAs with the other exonic and intronic sequences eliminated by spliceosomes (Figure 1D).

## Biogenesis and Mechanisms of Actions of circHIPK3

CircHIPK3 is an exonic circRNA, generated from the second exon of the homeodomain-interacting protein kinase 3 (*HIPK3*) gene that is located on chromosome 11p13 of humans (26). HIPK3 is one of the corepressors of homeodomain transcription factors (41). CircHIPK3 is conserved among humans, mice and other mammals (26, 42, 43).

**Figure 2:**
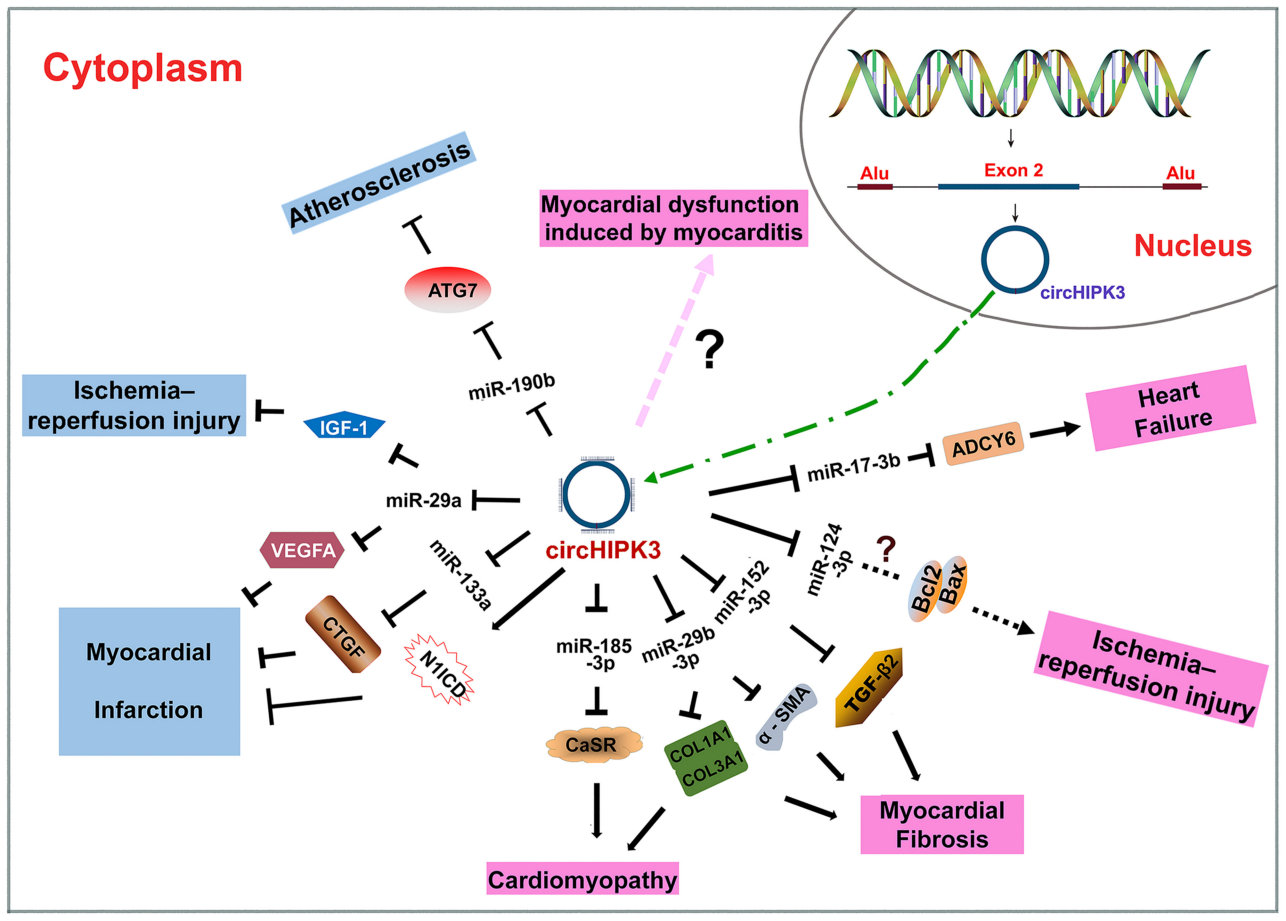
Biogenesis of circHIPK3 and the roles of circHIPK3 in CVD. CircHIPK3 is an ecRNA produced with the help of Alu repeats. CircHIPK3 is transported into the cytoplasm and functions as an miRNA sponge. CircHIPK3 can suppress the pathogenesis of atherosclerosis, myocardial injury, and myocardial infarction. CircHIPK3 can also promote the pathogenesis of cardiomyopathy, myocardial fibrosis, myocardial injury, myocardial dysfunction, and heart failure.

CircHIPK3 is produced through intron-pairing driven circularization with the help of Alu repeats (Figure 2) (26, 44). As an ecRNA, circHIPK3 can be transported into the cytoplasm and is mostly cytoplasm-located. CircHIPK3 is widely expressed in various tissues, such as the heart, lung and colon (26), consistent with its roles in CVD (32, 43–46), cancers (12, 27, 28, 47), and neuronal diseases (48, 49). CircHIPK3 mainly functions through sponging miRNAs (32, 34, 43, 44, 50). CircHIPK3 can be wrapped in exosomes to facilitate cell-to-cell communication (43, 51–53).

## CircHIPK3 and CVD

### Atherosclerosis

Atherosclerosis is an immune-inflammatory vascular disease that is usually chronic, and has a complex etiology (4, 54). Atherosclerosis is the underlying pathophysiological mechanism behind CAD and may lead to more severe heart diseases such as HF and MI (4, 54). Lipid metabolism disorders result in inflammatory signaling and significantly contribute to atherogenesis (55). Excessive low-density lipoprotein cholesterol (LDL-C) or oxidized low-density lipoprotein (ox-LDL) can promote atherogenesis (56). Autophagy is closely related to atherosclerosis, and its specific role is cell-type dependent (57). For example, autophagy in endothelial cells (EC) and vascular smooth muscle cells is protective against atherosclerosis (57). Various non-coding RNAs are involved in atherosclerosis, such as miRNAs (58, 59) and lncRNAs (60, 61). CircHIPK3 has also been found to be involved in atherosclerosis (46).

Wei et al. constructed an atherosclerotic model in mice with a high-fat diet (46). CircHIPK3 expression was decreased in the atherosclerotic mice, and autophagy was suppressed. Human umbilical vein endothelial cells (HUVECs) were treated with ox-LDL to construct an *in vitro* (outside the living experimental animals and in an artificial environment) atherosclerotic model. It has been established that miRNAs play an important role in EC autophagy (62, 63). CircHIPK3 was downregulated in ox-LDL treated HUVECs. Ox-LDL treatment could suppress autophagy. However, overexpression of circHIPK3 reverses the inhibitory effect of ox-LDL treatment on cell autophagy. CircHIPK3 could induce cell autophagy and improve atherosclerotic symptoms in an atherosclerotic cell model. Bioinformatics prediction analyses, dual luciferase assays, and RNA pull-down assays have shown that circHIPK3 could be a sponge of miR-190b. The effect of circHIPK3 on autophagy and atherosclerosis was inhibited by miR-190b overexpression (46). Further analyses showed that autophagy-related protein 7 (ATG7) was a direct target of miR-190b (64, 65). Previous studies have shown that deletion of ATGs (i.e., ATG5 and ATG7) can aggravate atherosclerosis (63, 66, 67). ATG7 downregulation reduced the autophagy level. CircHIPK3 knockdown led to decreased expression of ATG7. Knockdown of ATG7 and overexpression of miR-190b inhibited the function of circHIPK3 in autophagy. Therefore, circHIPK3 plays an antiatherosclerotic role by increasing autophagy via targeting the miR-190b-ATG7 pathway (46).

### Myocardial Infarction

MI is characterized by blockage of blood flow in coronary arteries, usually caused by blood clots forming on atherosclerotic plaques (20, 68). MI results in myocardial ischemia and damages the heart muscle (20, 68). A variety of non-coding RNAs function in the pathogenesis of MI (69–74). Recently, circHIPK3 has been shown to participate in MI (43, 53).

Si et al. found that circHIPK3 could promote CM proliferation and endothelial activation in the heart with MI through different mechanisms (43). The expression levels of circHIPK3 were significantly higher in fetal and neonatal hearts than in adult hearts, especially in the CMs of myocardial tissues. Gata4, a transcription factor responsible for CM proliferation and cardiac regeneration, could directly interact with the promoter of circHIPK3. Knockdown of Gata4 resulted in downregulation of circHIPK3. Overexpression of circHIPK3 promoted CM cell proliferation and suppressed apoptosis. Moreover, circHIPK3 overexpression could increase human coronary artery endothelial cell (HCAEC) proliferation and promote HCAEC tube formation and migration, implying a role of circHIPK3 in maintaining HCAEC function (43). In MI adult mice model, overexpression of circHIPK3 in the peri-infarcted area activates CM mitosis 14 days after MI. These findings suggest that circHIPK3 promotes CM regeneration. Furthermore, overexpression of circHIPK3 in the infarcted zone of MI mouse hearts could promote angiogenesis, reduce the scar size, and markedly elevate the myocardial perfusion score and cardiac pumping capacity. In P0 neonatal mouse hearts with MI, circHIPK3 knockdown significantly decreased the cardiac pumping capacity and increased the scar area (43). CircHIPK3 knockdown in the infarcted heart of neonatal MI mice reduced the proliferation and promoted the apoptosis of CMs. All of these results demonstrate that circHIPK3 could promote cardiac regeneration and improve cardiac function after MI. Subsequent experiments found that circHIPK3 could bind to miR-133a (a miRNA essential for heart development and protection) in HCAECs (43). MiR-133a was found to interact directly with connective tissue growth factor (CTGF), a growth factor involved in angiogenesis (75, 76). Overexpression of circHIPK3 significantly increased the level of CTGF, whereas miR-133a mimics attenuated this effect. In mouse hearts with MI, miR-133a overexpression significantly destroyed the improvement effect on angiogenesis induced by circHIPK3. These results suggest a regulatory role of the circHIPK3-miR-133a-CTGF axis in HCAEC function and angiogenesis. However, in CMs, circHIPK3 did not act as a miRNA sponge (43). RNA-Protein Interaction Prediction (RPISeq) and western blotting confirmed the interaction between circHIPK3 and Notch1 Intracellular Domain (N1ICD) protein, an important regulator of CM proliferation (77, 78). CircHIPK3 promoted N1ICD acetylation and elevated N1ICD stability, thereby preventing its degradation. CircHIPK3 knockdown reduced the level of N1ICD and inhibited CM proliferation (43). These findings indicate that circHIPK3 could promote CM proliferation by modulating N1ICD stability (43). In conclusion, circHIPK3 could activate endothelial cells through sponging miR-133a and promote CM proliferation by regulating N1ICD protein modification in MI hearts, suggesting that circHIPK3 may be a novel therapeutic target for the prevention of heart failure post-MI (43).

Wang et al. reported a role of exosomal circHIPK3 released from hypoxia-induced CMs in the regulation of cardiac angiogenesis after MI (53). Hypoxic exosomes (HPC-exos) (circHIPK3) released from CMs were delivered to the border area of MI. After 4 weeks, MI mice were found to have elevated cardiac pumping capacity and increased myocardial vascular density in the infarcted region. HPC-exos (circHIPK3) treatment relieved their symptoms of cardiac fibrosis. Under oxidative conditions, HPC-exos (circHIPK3) could promote angiogenesis by facilitating the migration and proliferation of cardiac endothelial cells. In addition, HPC-exos (circHIPK3) could induce tube formation. Hydrogen peroxide treatment decreased the expression of circHIPK3, whereas HPC-exo (circHIPK3) pretreatment significantly rescued the circHIPK3 level. In cardiac endothelial cells subjected to oxidative stress, overexpression of circHIPK3 in HPC-exos remarkably enhanced cell proliferation and migration ability. circHIPK3 was validated to bind to miR-29, which directly targets vascular endothelial growth factor A (VEGFA) (53), an angiogenesis-related factor (79). MiR-29a could significantly suppress the proliferation and migration of cardiac endothelial cells, inhibit tube formation, and decrease the number of branch points by targeting VEGFA (53). MiR-29a overexpression in cardiac endothelial cells could partly inhibit the HPC-exo-circHIPK3-induced promotion of tube formation and cell proliferation (53). In summary, HPC-exos (circHIPK3) plays a cardioprotective role by promoting angiogenesis and limiting the infarct size. CircHIPK3 can maintain the cardiac endothelial cell function post-MI via the miR-29a-VEGFA axis (53).

### Ischemia–Reperfusion Injury

Myocardial injury could have different etiology, such as HF, MI, and ischemia–reperfusion (I/R) (20). As a heart enriched circRNA, circHIPK3 has been found to be relevant to I/R injury caused by oxidative stress (50).

Several factors could contribute to I/R injury, including microcirculatory dysfunction and oxidative stress (50, 80). Cardiac microvascular endothelial cells (CMVECs) play a critical role in microcirculation and regulate cardiac function (81, 82). Exosomes are extracellular vesicles that participate in microcirculation. Exosomes usually function by transporting small bioactive molecules, including non-coding RNAs. Exosomes can be released from cardiomyocytes (CMs) under ischemic conditions (83). Wang et al. demonstrated that circHIPK3 could be packaged in exosomes (50). CMs treated with hypoxia secrete exosomes containing circHIPK3, and the exosomes are transported to CMVECs. CircHIPK3 is upregulated both in HPC-exosand in the CMVECs treated with HPC-exos. In contrast, circHIPK3 levels are significantly decreased in CMVECs pretreated with hydrogen peroxide. HPC-exos (containing circHIPK3) treatment could help CMVECs resist oxidative stress and rescue the levels of circHIPK3 in hydrogen peroxide-treated CMVECs, suggesting that circHIPK3 might protect CMVECs from oxidative damage. Luciferase reporter assays, AGO2 RNA immunoprecipitation (RIP), and FISH assays verified that circHIPK3 could interact with miR-29a. MiR-29a overexpression induced apoptosis and CMVEC injury, whereas downregulation of miR-29a protected CMVECs from oxidative stress injury and apoptosis. Further analyses showed that miR-29a could target insulin-like growth factor-1 (IGF-1), a multifunctional protein that inhibits apoptosis (84, 85). Upregulation of circHIPK3 in HPC-exos could increase the level of IGF-1 in CMVECs subjected to oxidative conditions by inhibiting the activity of miR-29a, thereby rescuing the dysfunction of CMVECs (50). In conclusion, circHIPK3 enclosed in HPC-exos might play a crucial role in CMVECs under oxidative stress via the miR-29a-IGF-1 axis to facilitate the repair of the damaged function of CMVECs.

Bai et al. illustrated the promoting role of circHIPK3 in I/R injury (32). An *in vitro* I/R injury model was induced by oxygen and glucose deprivation (OGD) and reperfusion (OGD/R) in human CM (HCM) cells. CircHIPK3 was upregulated in HCM cells with I/R injury. CCK-8 assays and flow cytometry revealed the suppressive effect of circHIPK3 on human CM cell proliferation and its promoting effect on apoptosis. Subsequent experiments showed that circHIPK3 could aggravate myocardial I/R by targeting miR-124-3p. Bax and Bcl-2, two apoptosis-related proteins (86), were found to have a dynamic expression in HCM cells along with the altered expression of circHIPK3 and miRNA-124-3p (32). However, the detailed underlying mechanisms are still unknown.

### Cardiomyopathy

Cardiomyopathy is a disease that could lead to HF due to an impaired ability of the heart to pump blood (87, 88). Due to different etiologies, there are different types of cardiomyopathy, such as hypertrophic cardiomyopathy (cardiac hypertrophy, blood flow blocked by stretched, and thickened heart muscles), dilated cardiomyopathy (loss of pumping power due to weakened heart muscles), ischemic cardiomyopathy (long-term myocardial ischemia), and diabetic cardiomyopathy (structural and functional abnormalities of the myocardium in diabetic patients). CircRNAs have been demonstrated to play critical roles in the pathogenesis of cardiomyopathy (20, 89, 90). Researchers have illuminated the function of circHIPK3 in hypertrophic cardiomyopathy (91) and diabetic cardiomyopathy (92).

In hypertrophic cardiomyopathy, the blood flow is decreased or blocked as the heart muscle become stretched and thick. Xu et al. reported that circHIPK3 expression was increased in the cardiac tissue of mice with cardiac hypertrophy (91). Knockdown of circHIPK3 alleviated cardiac hypertrophy symptoms both *in vivo* (animal models) and *in vitro*, indicating the promoting effect of circHIPK3 on cardiac hypertrophy. Further analyses showed that circHIPK3 could bind to miR-185-3p. Overexpression of circHIPK3 significantly reduced the level of miR-185-3p, while silencing of circHIPK3 elevated the expression of miR-185-3p. Calcium sensing receptor (CaSR) was the downstream target of miR-185-3p. CaSR has been shown to participate in cardiac physiology and pathophysiology (93–95). CircHIPK3 silencing resulted in a reduced level of CaSR, while overexpression of CaSR reversed the antihypertrophic effect of circHIPK3 silencing (91). Therefore, knockdown of circHIPK3 could inhibit hypertrophic cardiomyopathy through the miR-185-3p-CaSR axis (91).

As a serious complication of diabetes, diabetic cardiomyopathy might cause myocardial fibrosis, ventricular remodeling, and cardiac dysfunction (96, 97). Wang et al. elucidated the role of circHIPK3 in the pathogenesis of myocardial fibrosis in diabetic cardiomyopathy (92). CircHIPK3 was stably and highly expressed in the cytoplasm of cardiac fibroblasts (CFs). Treatment with high glucose concentrations increased the expression of circHIPK3 in CFs. In diabetic mice, circHIPK3 levels were elevated in the myocardium. Knockdown of circHIPK3 suppressed myocardial fibrosis and cardiac hypertrophy in diabetic mice. Left ventricular systolic function was impaired in diabetic mice, but could be improved by circHIPK3 silencing. Moreover, circHIPK3 silencing reduced the levels of fibrosis-associated proteins. CircHIPK3 was upregulated in CFs treated with angiotensin (Ang) II, which can induce the fibrotic phenotype (98). CircHIPK3 silencing repressed cell proliferation induced by Ang II. Bioinformatics prediction, dual luciferase reporter assays, and AGO2 RIP assays showed that circHIPK3 could target miR-29b-3p. Collagen type I alpha 1 (COL1A1) and collagen type III alpha 1 (COL3A1) were shown to be direct downstream targets of miR-29b-3p. Overexpression of circHIPK3 suppressed the inhibitory activity of miR-29b-3p on COL1A1 and COL3A1 (92). In general, circHIPK3 could promote myocardial fibrosis during diabetic cardiomyopathy by upregulating COL1A1/COL3A1 via suppressing miR-29b-3p (92).

### Myocardial Fibrosis

Myocardial fibrosis is a pathological process of CVD (20). In this process, CFs are activated to proliferate and differentiate into myofibroblasts (99). Then, numerous non-beating myofibroblasts replace the functional myocardium, resulting in myocardial dysfunction (99). CircHIPK3 has been identified to be abundantly expressed in CFs and to function in myocardial fibrosis (34, 44).

Ni et al. reported increased levels of circHIPK3 in Ang II-treated CFs and heart tissues (44). Silencing of circHIPK3 suppressed Ang II-induced CF proliferation and migration. RIP assays, bioinformatics analyses, and dual luciferase reporter assays were performed. The results demonstrated that circHIPK3 could bind to miR-29b-3p at two binding sites (44). MiR-29b-3p was also shown to interact with α-smooth muscle actin (α-SMA) and the COL1A1 and COL3A1 proteins, which are markers of myofibroblasts (100, 101). MiR-29b-3p overexpression inhibited CF cell migration and reduced the protein expression of α-SMA, COL1A1, and COL3A1 protein. In summary, circHIPK3 might stimulate the progression of cardiac fibrosis and attenuate diastolic function by sponging miR-29b-3p (44).

Liu et al. illuminated the role of circHIPK3 in cardiac fibrosis under hypoxia (34). The expression of circHIPK3 was significantly increased in CFs treated with hypoxia. CircHIPK3 could strongly promote the proliferation, migration, and phenotypic switching of CFs under hypoxia. Further analyses indicated that circHIPK3 could sponge miR-152-3p which might inhibit CF proliferation and cause phenotypic changes. MiR-152-3p was found to interact with transforming growth factor β (TGF-β2). Downregulation of circHIPK3 could result in decreased levels of TGF-β2 by upregulating miR-152-3p (34). Therefore, circHIPK3 might participate in the development of cardiac fibrosis through the miR-152-3p-TGF-β2 axis (34).

### Heart Failure

Heart failure (HF) is a serious cardiac disease with symptoms resulting from a structural and functional cardiac abnormality (102). The clinical symptoms are accompanied by increased natriuretic peptide levels or objective diagnostic evidence (imaging or hemodynamic measurement) of cardiogenic pulmonary or systemic congestion (102). Severe HF can lead to death, and therefore, HF should be detected and treated as early as possible. CircHIPK3 has been identified to enhance the effect of adrenaline in treating HF (103).

β-blocker is an efficient intervention drug for HF and has been found to function through blocking the activation of the β-adrenergic receptor (β-AR) (104). β-AR can improve cardiac function in the short term, but it increases the mortality rate in the long term (104). Calcium plays an important role in HF (105, 106). Deng et al. found that the level of circHIPK3 was remarkably increased in mouse hearts with HF post-MI (103). Bioinformatics analyses showed that circHIPK3 might participate in adrenergic signaling or the calcium pathway. CircHIPK3 overexpression increased the calcium concentration in cytoplasm, whereas the downregulation of circHIPK3 reduced the calcium concentration. Further analyses revealed that circHIPK3 could interact with miR-17-3p to regulate the calcium distribution. Adenylated cyclase type 6 (ADCY6), an isoform of the calcium-inhibited family (107), was shown to be a target of miR-17-3p. Overexpression of circHIPK3 upregulated the level of ADCY6. This effect could be suppressed by miR-17-3p. *In vitro* studies of neonatal mouse CMs indicated that circHIPK3 might function through the miR-17-3p-ADCY6 axis. Adrenaline has been shown to upregulate the level of circHIPK3 through cAMP responsive element-binding protein 1 (CREB1) (108), a key transcription factor that can be activated by various growth factors and stress signals (108). Downregulation of circHIPK3 *in vivo* alleviated cardiac fibrosis and heart remodeling post-MI, thereby maintaining heart function (103). Therefore, circHIPK3 could assist the function of adrenaline in cardiomyocytes via the miR-17-3p-ADCY6 axis.

### Myocardial Dysfunction Induced by Myocarditis

Myocardial dysfunction is a typical type of cardiac dysfunction that results in the proliferation of inflammatory lesions in the myocardium (109). There are several causes of myocardial dysfunction, such as infectious pathogens and toxic and hypersensitivity reactions (110, 111). circHIPK3 has been shown to play a role in regulating myocardial dysfunction caused by myocarditis (112).

Fan et al. found that knockdown of circHIPK3 could elevate heart rate and left ventricle ejection fraction, and significantly reduce the expression of heart damage markers, demonstrating that knockdown of circHIPK3 can repress heart damage and inhibit CM apoptosis (112). In addition, knockdown of circHIPK3 also effectively attenuated oxidative stress and inflammation *in vivo*. The level of circHIPK3 was significantly increased when exposed to lipopolysaccharide (LPS) *in vivo* and *in vitro*. LPS can induce apoptosis, inflammatory events and oxidative damage, resulting in serious tissue damage. Knockdown of circHIPK3 partly reversed these damaging effects and protected the myocardium. In general, the downregulation of circHIPK3 could effectively ameliorate the symptoms of LPS-induced myocarditis (112).

The result of this article is contrary to that of Wang et al. and Si et al. on the effect of circHIPK3 in cardiac dysfunction (43, 50). We speculate that the contradicting results may be due to differences in the methods and sample sizes.

## Concluding Remarks

CircHIPK3 is an ecRNA that is conserved among many species. CircHIPK3 is multifunctional; it has been shown to participate in various physiological and pathological processes. CircHIPK3 has several characteristics, including high conservation, high stability, extracellular secretion ability and dynamic expression under different physiological and pathological conditions. In the past 5 years, many studies on the role of circHIPK3 in various diseases have been reported. CircHIPK3 has been demonstrated to participate in the occurrence and development of CVD (Table 1). The sponging of miRNAs is the primary mechanism of action of circHIPK3. The circHIPK3-miRNA-protein signaling pathway allows circHIPK3 to function in the pathogenesis of different CVD patterns via various miRNA-protein axes (Figure 2). Therefore, circHIPK3 could have clinical applications in the diagnosis and treatment of CVD.

**Table 1:** The mechanisms of circHIPK3 in CVD.

**CVD**	**Subjects**	**Expression**	**Regulatory Mechanism**	**Effect**	**References**
Atherosclerosis	Atherosclerosis mouse	Downregulated	circHIPK3-miR-190b-ATG7	Suppression	(46)
Myocardial infarction	MI mouse, HCAEC, CMs	–	Gata4-circHIPK3-miR-133a-CTGF	Suppression	(43)
			circHIPK3-N1ICD		
	MI mouse, cardiac endothelial cells	Downregulated	circHIPK3-miR-29a-VEGFA	Suppression	(53)
Ischemia-reperfusion injury	Hypoxia treated-CMVEC	Downregulated	circHIPK3-miR-29a-IGF-1	Suppression	(50)
	HCM cells with I/R injury	Upregulated	circHIPK3-miR-124-3p–?–Bax/Bcl-2	Promotion	(32)
Cardiomyopathy	Hypertrophic cardiomyopathy mouse	Upregulated	circHIPK3-miR-185-3p-CaSR	Promotion	(91)
	Diabetic cardiomyopathy mouse, CFs	Upregulated	circHIPK3-miR-29b-3p-COL1A1/COL3A1	Promotion	(92)
Myocardial fibrosis	CFs and heart tissues	Upregulated	circHIPK3-miR-29b-3p-α-SMA/COL1A1/COL3A1	Promotion	(44)
	CFs under hypoxia	Upregulated	circHIPK3- miR-152-3p-TGFβ2	Promotion	(34)
Heart failure	Mouse heart with HF post MI	Upregulated	circHIPK3-miR-17-3p-ADCY6	Promotion	(103)
Myocardial dysfunction induced by myocarditis	CMs	Upregulated	–	Promotion	(112)

However, several gaps in knowledge and limitations should be addressed. First, the sample sizes in most reports were relatively small. The insufficient samples might have led to inaccurate results, which could explain the conflicting findings among studies. Therefore, further studies with a larger sample size are needed. Second, there has been substantial research on circHIPK3, but the underlying mechanisms of circHIPK3 in many diseases are still unclear. Therefore, more investigation and efforts should be made to unveil the details of the mechanisms. Third, the existing forms of circHIPK3 in different pathological processes need to be explored. CircRNAs can exist as free molecules or be confined inside extracellular vesicles (e.g., exosomes), which would definitely affect their function.

In summary, circHIPK3 is widely involved in the development of CVD. It functions through sponging miRNAs. The current findings suggest potential clinical uses of cirHIPK3 in the prognosis and treatment of CVDs.

## Author Contributions

LZ drafted the manuscript. YW and FY edited the manuscript. HG and XL revised the manuscript. PL and LZ conceived the idea of the review and made the final proof reading. All authors read and approved the final manuscript.

## Funding

This work was supported by the National Natural Science Foundation of China (Grant No. 91849209) and Shandong Provincial Natural Science Foundation, China (Grant No. ZR2020QH016).

## Conflict of Interest

The authors declare that the research was conducted in the absence of any commercial or financial relationships that could be construed as a potential conflict of interest.

## Publisher's Note

All claims expressed in this article are solely those of the authors and do not necessarily represent those of their affiliated organizations, or those of the publisher, the editors and the reviewers. Any product that may be evaluated in this article, or claim that may be made by its manufacturer, is not guaranteed or endorsed by the publisher.

## References

[1] Chen X Ba Y Ma L Cai X Yin Y Wang K. Characterization of microRNAs in serum: a novel class of biomarkers for diagnosis of cancer and other diseases. Cell Res. (2008) 18:997–1006. 10.1038/cr.2008.282 18766170

[2] Hu J Kong M Ye Y Hong S Cheng L Jiang L. Serum miR-206 and other muscle-specific microRNAs as non-invasive biomarkers for duchenne muscular dystrophy. J Neurochem. (2014) 129:877–83. 10.1111/jnc.12662 24460924

[3] Yang ZG Guo XB Li GM Shi YL Li LP. Long noncoding RNAs as potential biomarkers in gastric cancer: opportunities and challenges. Cancer Lett. (2016) 371:62–70. 10.1016/j.canlet.2015.11.011 26577810

[4] Zhang L Zhang Y Zhao YF Wang Y Ding H Xue S. Circulating miRNAs as biomarkers for early diagnosis of coronary artery disease. Exp Opin Ther Pat. (2018) 28:591–601. 10.1080/13543776.2018.1503650 30064285

[5] Zhang Y Zhang L Wang Y Ding H Xue S Yu H. KCNQ1OT1, HIF1A-AS2 and APOA1-AS are promising novel biomarkers for diagnosis of coronary artery disease. Clin Exp Pharmacol Physiol. (2019) 46:635–42. 10.1111/1440-1681.13094 30941792

[6] Zhang L Zhang Y Xue S Ding H Wang Y Qi HZ. Clinical significance of circulating microRNAs as diagnostic biomarkers for coronary artery disease. J Cell Mol Med. (2020) 24:1146–50. 10.1111/jcmm.14802 31709737 PMC6933363

[7] Kolakofsky D. Isolation and characterization of sendai virus DI-RNAs. Cell. (1976) 8:547–55. 10.1016/0092-8674(76)90223-3 182384

[8] Nigro JM Cho KR Fearon ER Kern SE Ruppert JM Oliner JD. Scrambled exons. Cell. (1991) 64:607–13. 10.1016/0092-8674(91)90244-S 1991322

[9] Cocquerelle C Daubersies P Majerus MA Kerckaert JP Bailleul B. Splicing with inverted order of exons occurs proximal to large introns. EMBO J. (1992) 11:1095–8. 10.1002/j.1460-2075.1992.tb05148.x 1339341 PMC556550

[10] Capel B Swain A Nicolis S Hacker A Walter M Koopman P. Circular transcripts of the testis-determining gene sry in adult mouse testis. Cell. (1993) 73:1019–30. 10.1016/0092-8674(93)90279-Y 7684656

[11] Yang Y Gao X Zhang M Yan S Sun C Xiao F. Novel role of FBXW7 circular RNA in repressing glioma tumorigenesis. J Natl Cancer Inst. (2018) 110:304–15. 10.1093/jnci/djx166 28903484 PMC6019044

[12] Huang HR Wei L Qin T Yang N Li ZD Xu ZY. Circular RNA ciRS-7 triggers the migration and invasion of esophageal squamous cell carcinoma via miR-7/KLF4 and NF-kappa B signals. Cancer Biol Ther. (2019) 20:73–80. 10.1080/15384047.2018.1507254 30207835 PMC6343722

[13] Liang WC Wong CW Liang PP Shi M Cao Y Rao ST. Translation of the circular RNA circ-catenin promotes liver cancer cell growth through activation of the Wnt pathway. Genome Biol. (2019) 20:84. 10.1186/s13059-019-1685-4 31027518 PMC6486691

[14] Zheng X Chen L Zhou Y Wang Q Zheng Z Xu B. A novel protein encoded by a circular RNA circPPP1R12A promotes tumor pathogenesis and metastasis of colon cancer via Hippo-YAP signaling. Mol Cancer. (2019) 18:47. 10.1186/s12943-019-1010-6 30925892 PMC6440158

[15] Zhang L Wang Y Zhang Y Zhao Y Li P. Pathogenic mechanisms and the potential clinical value of circFoxo3 in cancers. Mol Ther Nucleic Acids. (2021) 23:908–17. 10.1016/j.omtn.2021.01.010 33614239 PMC7868936

[16] Li H Xu JD Fang XH Zhu JN Yang J Pan R. Circular RNA circRNA_000203 aggravates cardiac hypertrophy via suppressing miR-26b-5p and miR-140-3p binding to Gata4. Cardiovasc Res. (2019) 116:1323–34. 10.1093/cvr/cvz215 31397837 PMC7243276

[17] Sun Y Zhang SL Yue MM Li Y Bi J Liu HR. Angiotensin II inhibits apoptosis of mouse aortic smooth muscle cells through regulating the circNRG-1/miR-193b-5p/NRG-1 axis. Cell Death Dis. (2019) 10:362. 10.1038/s41419-019-1590-5 31043588 PMC6494886

[18] Zhou LY Zhai M Huang Y Xu S An T Wang YH. The circular RNA ACR attenuates myocardial ischemia/reperfusion injury by suppressing autophagy via modulation of the Pink1/ FAM65B pathway. Cell Death Differ. (2019) 26:1299–315. 10.1038/s41418-018-0206-4 30349076 PMC6748144

[19] Lim TB Lavenniah A Foo RS. Circles in the heart and cardiovascular system. Cardiovasc Res. (2020) 116:269–78. 10.1093/cvr/cvz227 31552406

[20] Zhang L Zhang Y Wang Y Zhao Y Ding H Li P. Circular RNAs: functions and clinical significance in cardiovascular disease. Front Cell Dev Biol. (2020) 8:584051. 10.3389/fcell.2020.584051 33134301 PMC7550538

[21] Hansen TB Jensen TI Clausen BH Bramsen JB Finsen B Damgaard CK. Natural RNA circles function as efficient microRNA sponges. Nature. (2013) 495:384–88. 10.1038/nature11993 23446346

[22] Ashwal-Fluss R Meyer M Pamudurti NR Ivanov A Bartok O Hanan M. circRNA biogenesis competes with pre-mRNA splicing. Mol Cell. (2014) 56:55–66. 10.1016/j.molcel.2014.08.019 25242144

[23] Zhang Y Zhang XO Chen T Xiang JF Yin QF Xing YH. Circular intronic long noncoding RNAs. Mol Cell. (2013) 51:792–806. 10.1016/j.molcel.2013.08.017 24035497

[24] Li ZY Huang C Bao C Chen L Lin M Wang XL. Exon-intron circular RNAs regulate transcription in the nucleus. Nat Struc Mol Biol. (2015) 22:256–64. 10.1038/nsmb.2959 25664725

[25] Yang Y Fan X Mao M Song X Wu P Zhang Y. Extensive translation of circular RNAs driven by N(6)-methyladenosine. Cell Res. (2017) 27:626–41. 10.1038/cr.2017.31 28281539 PMC5520850

[26] Zheng Q Bao C Guo W Li S Chen J Chen B. Circular RNA profiling reveals an abundant circHIPK3 that regulates cell growth by sponging multiple miRNAs. Nat Commun. (2016) 7:11215. 10.1038/ncomms11215 27050392 PMC4823868

[27] Zeng K Chen X Xu M Liu X Hu X Xu T. CircHIPK3 promotes colorectal cancer growth and metastasis by sponging miR-7. Cell Death Dis. (2018) 9:417. 10.1038/s41419-018-0454-8 29549306 PMC5856798

[28] Jin Y Che X Qu X Li X Lu W Wu J. CircHIPK3 promotes metastasis of gastric cancer via miR-653-5p/miR-338-3p-NRP1 axis under a long-term hypoxic microenvironment. Front Oncol. (2020) 10:1612. 10.3389/fonc.2020.01612 32903845 PMC7443574

[29] Zhu X Wang X Wang Y Zhao Y. The regulatory network among CircHIPK3, LncGAS5, and miR-495 promotes Th2 differentiation in allergic rhinitis. Cell Death Dis. (2020) 11:216. 10.1038/s41419-020-2394-3 32242002 PMC7118158

[30] Qiu Z Wang Y Liu W Li C Zhao R Long X. CircHIPK3 regulates the autophagy and apoptosis of hypoxia/reoxygenation-stimulated cardiomyocytes via the miR-20b-5p/ATG7 axis. Cell Death Discov. (2021) 7:64. 10.1038/s41420-021-00448-6 33824287 PMC8024346

[31] Chen G Shi Y Liu M Sun J. circHIPK3 regulates cell proliferation and migration by sponging miR-124 and regulating AQP3 expression in hepatocellular carcinoma. Cell Death Dis. (2018) 9:175. 10.1038/s41419-017-0204-3 29415990 PMC5833724

[32] Bai M Pan CL Jiang GX Zhang YM Zhang Z. CircHIPK3 aggravates myocardial ischemia-reperfusion injury by binding to miRNA-124-3p. Eur Rev Med Pharmacol Sci. (2019) 23:10107–14. 10.26355/eurrev_201911_19580 31799682

[33] He X Ou C. CircRNA circHIPK3: a novel therapeutic target for angiotensin II-induced cardiac fibrosis. Int J Cardiol. (2020) 312:98. 10.1016/j.ijcard.2020.03.034 32197828

[34] Liu W Wang Y Qiu Z Zhao R Liu Z Chen W. CircHIPK3 regulates cardiac fibroblast proliferation, migration and phenotypic switching through the miR-152-3p/TGF-beta2 axis under hypoxia. PeerJ. (2020) 8:e9796. 10.7717/peerj.9796 32904464 PMC7453924

[35] Wen J Liao J Liang J Chen XP Zhang B Chu L. Circular RNA HIPK3: a key circular RNA in a variety of human cancers. Front Oncol. (2020) 10:773. 10.3389/fonc.2020.00773 32500032 PMC7242753

[36] Zhang XO Wang HB Zhang Y Lu XH Chen LL Yang L. Complementary sequence-mediated exon circularization. Cell. (2014) 159:134–47. 10.1016/j.cell.2014.09.001 25242744

[37] Memczak S Jens M Elefsinioti A Torti F Krueger J Rybak A. Circular RNAs are a large class of animal RNAs with regulatory potency. Nature. (2013) 495:333–8. 10.1038/nature11928 23446348

[38] Jeck WR Sorrentino JA Wang K Slevin MK Burd CE Liu JZ. Circular RNAs are abundant, conserved, and associated with ALU repeats. Rna. (2013) 19:141–57. 10.1261/rna.035667.112 23249747 PMC3543092

[39] Jeck WR Sharpless NE. Detecting and characterizing circular RNAs. Nat Biotechnol. (2014) 32:453–61. 10.1038/nbt.2890 24811520 PMC4121655

[40] Conn SJ Pillman KA Toubia J Conn VM Salmanidis M Phillips CA. The RNA binding protein quaking regulates formation of circRNAs. Cell. (2015) 160:1125–34. 10.1016/j.cell.2015.02.014 25768908

[41] Conte A Pierantoni GM. Update on the regulation of HIPK1, HIPK2 and HIPK3 protein kinases by microRNAs. Microrna. (2018) 7:178–86. 10.2174/2211536607666180525102330 29793420

[42] Chen B Yu J Guo L Byers MS Wang Z Chen X. Circular RNA circHIPK3 promotes the proliferation and differentiation of chicken myoblast cells by sponging miR-30a-3p. Cells. (2019) 8:177. 10.3390/cells8020177 30791438 PMC6406597

[43] Si X Zheng H Wei G Li M Li W Wang H. circRNA hipk3 induces cardiac regeneration after myocardial infarction in mice by binding to Notch1 and miR-133a. Mol Ther Nucleic Acids. (2020) 21:636–55. 10.1016/j.omtn.2020.06.024 32736292 PMC7393325

[44] Ni H Li W Zhuge Y Xu S Wang Y Chen Y. Inhibition of circHIPK3 prevents angiotensin II-induced cardiac fibrosis by sponging miR-29b-3p. Int J Cardiol. (2019) 292:188–96. 10.1016/j.ijcard.2019.04.006 30967276

[45] Lin J Feng X Zhang J. Circular RNA circHIPK3 modulates the proliferation of airway smooth muscle cells by miR-326/STIM1 axis. Life Sci. (2020) 255:117835. 10.1016/j.lfs.2020.117835 32450169

[46] Wei MY Lv RR Teng Z. Circular RNA circHIPK3 as a novel circRNA regulator of autophagy and endothelial cell dysfunction in atherosclerosis. Eur Rev Med Pharmacol Sci. (2020) 24:12849–58. 10.26355/eurrev_202012_24187 33378035

[47] Ke Z Xie F Zheng C Chen D. CircHIPK3 promotes proliferation and invasion in nasopharyngeal carcinoma by abrogating miR-4288-induced ELF3 inhibition. J Cell Physiol. (2019) 234:1699–706. 10.1002/jcp.27041 30070690

[48] Hu D Zhang Y. Circular RNA HIPK3 promotes glioma progression by binding to miR-124-3p. Gene. (2019) 690:81–9. 10.1016/j.gene.2018.11.073 30576808

[49] Zhao J Qi X Bai J Gao X Cheng L. A circRNA derived from linear HIPK3 relieves the neuronal cell apoptosis in spinal cord injury via ceRNA pattern. Biochem Biophys Res Commun. (2020) 528:359–67. 10.1016/j.bbrc.2020.02.108 32247616

[50] Wang Y Zhao R Liu W Wang Z Rong J Long X. Exosomal circHIPK3 released from hypoxia-pretreated cardiomyocytes regulates oxidative damage in cardiac microvascular endothelial cells via the miR-29a/IGF-1 pathway. Oxid Med Cell Longev. (2019) 2019:7954657. 10.1155/2019/7954657 31885817 PMC6915129

[51] Lasda E Parker R. Circular RNAs co-precipitate with extracellular vesicles: a possible mechanism for circrna clearance. PLoS ONE. (2016) 11:e0148407. 10.1371/journal.pone.0148407 26848835 PMC4743949

[52] Talman V Kivela R. Cardiomyocyte-endothelial cell interactions in cardiac remodeling and regeneration. Front Cardiovasc Med. (2018) 5:101. 10.3389/fcvm.2018.00101 30175102 PMC6108380

[53] Wang Y Zhao R Shen C Liu W Yuan J Li C. Exosomal CircHIPK3 released from hypoxia-induced cardiomyocytes regulates cardiac angiogenesis after myocardial infarction. Oxid Med Cell Longev. (2020) 2020:8418407. 10.1155/2020/8418407 32733638 PMC7376438

[54] Falk E. Pathogenesis of atherosclerosis. J Am Coll Cardiol. (2006) 47:C7–12. 10.1016/j.jacc.2005.09.068 16631513

[55] Schaftenaar F Frodermann V Kuiper J Lutgens E. Atherosclerosis: the interplay between lipids and immune cells. Curr Opin Lipidol. (2016) 27:209–15. 10.1097/MOL.0000000000000302 27031276

[56] Libby P Buring JE Badimon L Hansson GK Deanfield J Bittencourt MS. Atherosclerosis. Nat Rev Dis Primers. (2019) 5:56. 10.1038/s41572-019-0106-z 31420554

[57] Henderson JM Weber C Santovito D. Beyond self-recycling: cell-specific role of autophagy in atherosclerosis. Cells. (2021) 10:625. 10.3390/cells10030625 33799835 PMC7998923

[58] Feinberg MW Moore KJ. MicroRNA regulation of atherosclerosis. Circ Res. (2016) 118:703–20. 10.1161/CIRCRESAHA.115.306300 26892968 PMC4762069

[59] Di Gregoli K Mohamad Anuar NN Bianco R White SJ Newby AC George SJ. MicroRNA-181b controls atherosclerosis and aneurysms through regulation of TIMP-3 and elastin. Circ Res. (2017) 120:49–65. 10.1161/CIRCRESAHA.116.309321 27756793 PMC5214094

[60] Ye ZM Yang S Xia YP Hu RT Chen S Li BW. LncRNA MIAT sponges miR-149-5p to inhibit efferocytosis in advanced atherosclerosis through CD47 upregulation. Cell Death Dis. (2019) 10:138. 10.1038/s41419-019-1409-4 30755588 PMC6372637

[61] Josefs T Boon RA. The long non-coding road to atherosclerosis. Curr Atheroscler Rep. (2020) 22:55. 10.1007/s11883-020-00872-6 32772181 PMC7415749

[62] Pankratz F Hohnloser C Bemtgen X Jaenich C Kreuzaler S Hoefer I. MicroRNA-100 suppresses chronic vascular inflammation by stimulation of endothelial autophagy. Circ Res. (2018) 122:417–32. 10.1161/CIRCRESAHA.117.311428 29208678

[63] Santovito D Egea V Bidzhekov K Natarelli L Mourao A Blanchet X. Noncanonical inhibition of caspase-3 by a nuclear microRNA confers endothelial protection by autophagy in atherosclerosis. Sci Transl Med. (2020) 12:eaaz2294. 10.1126/scitranslmed.aaz2294 32493793

[64] Mizushima N Levine B. Autophagy in mammalian development and differentiation. Nat Cell Biol. (2010) 12:823–30. 10.1038/ncb0910-823 20811354 PMC3127249

[65] Mizushima N Yoshimori T Ohsumi Y. The role of Atg proteins in autophagosome formation. Annu Rev Cell Dev Biol. (2011) 27:107–32. 10.1146/annurev-cellbio-092910-154005 21801009

[66] Torisu K Singh KK Torisu T Lovren F Liu J Pan Y. Intact endothelial autophagy is required to maintain vascular lipid homeostasis. Aging Cell. (2016) 15:187–91. 10.1111/acel.12423 26780888 PMC4717267

[67] Vion AC Kheloufi M Hammoutene A Poisson J Lasselin J Devue C. Autophagy is required for endothelial cell alignment and atheroprotection under physiological blood flow. Proc Natl Acad Sci USA. (2017) 114:E8675–84. 10.1073/pnas.1702223114 28973855 PMC5642679

[68] Lu L Liu M Sun R Zheng Y Zhang P. Myocardial infarction: symptoms and treatments. Cell Biochem Biophys. (2015) 72:865–7. 10.1007/s12013-015-0553-4 25638347

[69] Boon RA Dimmeler S. MicroRNAs in myocardial infarction. Nat Rev Cardiol. (2015) 12:135–42. 10.1038/nrcardio.2014.207 25511085

[70] Jiang X Ning Q. The emerging roles of long noncoding RNAs in common cardiovascular diseases. Hypertens Res. (2015) 38:375–9. 10.1038/hr.2015.26 25762413

[71] Uchida S Dimmeler S. Long noncoding RNAs in cardiovascular diseases. Circ Res. (2015) 116:737–50. 10.1161/CIRCRESAHA.116.302521 25677520

[72] Han F Chen Q Su J Zheng A Chen K Sun S. MicroRNA-124 regulates cardiomyocyte apoptosis and myocardial infarction through targeting Dhcr24. J Mol Cell Cardiol. (2019) 132:178–88. 10.1016/j.yjmcc.2019.05.007 31100313

[73] Trembinski DJ Bink DI Theodorou K Sommer J Fischer A van Bergen A. Aging-regulated anti-apoptotic long non-coding RNA sarrah augments recovery from acute myocardial infarction. Nat Commun. (2020) 11:2039. 10.1038/s41467-020-15995-2 32341350 PMC7184724

[74] Zhang L Ding H Zhang Y Wang Y Zhu W Li P. Circulating MicroRNAs: biogenesis and clinical significance in acute myocardial infarction. Front Physiol. (2020) 11:1088. 10.3389/fphys.2020.01088 33013463 PMC7494963

[75] Duan LJ Qi J Kong XJ Huang T Qian XQ Xu D. MiR-133 modulates TGF-beta1-induced bladder smooth muscle cell hypertrophic and fibrotic response: implication for a role of microRNA in bladder wall remodeling caused by bladder outlet obstruction. Cell Signal. (2015) 27:215–27. 10.1016/j.cellsig.2014.11.001 25451078

[76] Yang L Hou J Cui XH Suo LN Lv YW. MiR-133b regulates the expression of CTGF in epithelial-mesenchymal transition of ovarian cancer. Eur Rev Med Pharmacol Sci. (2017) 21:5602–9. 10.26355/eurrev_201712_14001 29271992

[77] Metrich M Bezdek Pomey A Berthonneche C Sarre A Nemir M Pedrazzini T. Jagged1 intracellular domain-mediated inhibition of Notch1 signalling regulates cardiac homeostasis in the postnatal heart. Cardiovasc Res. (2015) 108:74–86. 10.1093/cvr/cvv209 26249804 PMC4571837

[78] Collesi C Felician G Secco I Gutierrez MI Martelletti E Ali H. Reversible Notch1 acetylation tunes proliferative signalling in cardiomyocytes. Cardiovasc Res. (2018) 114:103–22. 10.1093/cvr/cvx228 29186476

[79] Ferrara N. Vascular endothelial growth factor: basic science and clinical progress. Endocr Rev. (2004) 25:581–611. 10.1210/er.2003-0027 15294883

[80] Yu F Liu Y Xu J. Pro-BDNF contributes to hypoxia/reoxygenation injury in myocardial microvascular endothelial cells: roles of receptors p75(NTR) and Sortilin and activation of JNK and caspase 3. Oxid Med Cell Longev. (2018) 2018:3091424. 10.1155/2018/3091424 30046375 PMC6038493

[81] Brutsaert DL. Cardiac endothelial-myocardial signaling: its role in cardiac growth, contractile performance, and rhythmicity. Physiol Rev. (2003) 83:59–115. 10.1152/physrev.00017.2002 12506127

[82] Fujita Y Kawamoto A. Stem cell-based peripheral vascular regeneration. Adv Drug Deliv Rev. (2017) 120:25–40. 10.1016/j.addr.2017.09.001 28912015

[83] Ribeiro-Rodrigues TM Laundos TL Pereira-Carvalho R Batista-Almeida D Pereira R Coelho-Santos V. Exosomes secreted by cardiomyocytes subjected to ischaemia promote cardiac angiogenesis. Cardiovasc Res. (2017) 113:1338–50. 10.1093/cvr/cvx118 28859292

[84] Troncoso R Ibarra C Vicencio JM Jaimovich E Lavandero S. New insights into IGF-1 signaling in the heart. Trends Endocrinol Metab. (2014) 25:128–37. 10.1016/j.tem.2013.12.002 24380833

[85] Wang L Niu X Hu J Xing H Sun M Wang J. After myocardial ischemia-reperfusion, miR-29a, and let7 could affect apoptosis through regulating IGF-1. Biomed Res Int. (2015) 2015:245412. 10.1155/2015/245412 26844226 PMC4710957

[86] Miyashita T Krajewski S Krajewska M Wang HG Lin HK Liebermann DA. Tumor-Suppressor P53 is a regulator of Bcl-2 and bax gene-expression *in-vitro* and *in-vivo*. Oncogene. (1994) 9:1799–805. 8183579

[87] Nakamura M Sadoshima J. Mechanisms of physiological and pathological cardiac hypertrophy. Nat Rev Cardiol. (2018) 15:387–407. 10.1038/s41569-018-0007-y 29674714

[88] Oldfield CJ Duhamel TA Dhalla NS. Mechanisms for the transition from physiological to pathological cardiac hypertrophy. Can J Physiol Pharmacol. (2020) 98:74–84. 10.1139/cjpp-2019-0566 31815523

[89] Lim TB Aliwarga E Luu TDA Li YP Ng SL Annadoray L. Targeting the highly abundant circular RNA circSlc8a1 in cardiomyocytes attenuates pressure overload induced hypertrophy. Cardiovasc Res. (2019) 115:1998–2007. 10.1093/cvr/cvz130 31114845

[90] Li H Xu JD Fang XH Zhu JN Yang J Pan R. Circular RNA circRNA_000203 aggravates cardiac hypertrophy via suppressing miR-26b-5p and miR-140-3p binding to Gata4. Cardiovasc Res. (2020) 116:1323–34. 31397837 10.1093/cvr/cvz215 PMC7243276

[91] Xu X Wang J Wang X. Silencing of circHIPK3 inhibits pressure overload-induced cardiac hypertrophy and dysfunction by sponging miR-185-3p. Drug Des Devel Ther. (2020) 14:5699–710. 10.2147/DDDT.S245199 33402817 PMC7778681

[92] Wang W Zhang S Xu L Feng Y Wu X Zhang M. Involvement of circHIPK3 in the pathogenesis of diabetic cardiomyopathy in mice. Diabetologia. (2021) 64:681–92. 10.1007/s00125-020-05353-8 33398455

[93] Brancaccio D Cozzolino M. [Cardiovascular effects of VDR and CaSR activation]. G Ital Nefrol. (2009) 26 (Suppl. 49):S18–22. 19941274

[94] Toka HR Pollak MR. The role of the calcium-sensing receptor in disorders of abnormal calcium handling and cardiovascular disease. Curr Opin Nephrol Hypertens. (2014) 23:494–501. 10.1097/MNH.0000000000000042 24992569

[95] Diaz-Soto G Rocher A Garcia-Rodriguez C Nunez L Villalobos C. The calcium-sensing receptor in health and disease. Int Rev Cell Mol Biol. (2016) 327:321–69. 10.1016/bs.ircmb.2016.05.004 27692178

[96] Jia GH Hill MA Sowers JR. Diabetic cardiomyopathy: an update of mechanisms contributing to this clinical entity. Circu Res. (2018) 122:624–38. 10.1161/CIRCRESAHA.117.311586 29449364 PMC5819359

[97] Marwick TH Ritchie R Shaw JE Kaye D. Implications of underlying mechanisms for the recognition and management of diabetic cardiomyopathy. J Am Coll Cardiol. (2018) 71:339–51. 10.1016/j.jacc.2017.11.019 29348027

[98] Ding J Tang Q Luo B Zhang L Lin L Han L. Klotho inhibits angiotensin II-induced cardiac hypertrophy, fibrosis, and dysfunction in mice through suppression of transforming growth factor-beta1 signaling pathway. Eur J Pharmacol. (2019) 859:172549. 10.1016/j.ejphar.2019.172549 31325434

[99] Gyongyosi M Winkler J Ramos I Do QT Firat H McDonald K. Myocardial fibrosis: biomedical research from bench to bedside. Eur J Heart Fail. (2017) 19:177–91. 10.1002/ejhf.696 28157267 PMC5299507

[100] Hinz B Celetta G Tomasek JJ Gabbiani G Chaponnier C. Alpha-smooth muscle actin expression upregulates fibroblast contractile activity. Mol Biol Cell. (2001) 12:2730–41. 10.1091/mbc.12.9.2730 11553712 PMC59708

[101] Bella J Hulmes DJ. Fibrillar collagens. Subcell Biochem. (2017) 82:457–90. 10.1007/978-3-319-49674-0_14 28101870

[102] Bozkurt B Coats AJS Tsutsui H Abdelhamid CM Adamopoulos S Albert N. Universal definition and classification of heart failure: a report of the heart failure society of america, heart failure association of the European society of cardiology, japanese heart failure society and writing committee of the universal definition of heart failure: endorsed by the Canadian heart failure society, heart failure association of india, cardiac society of Australia and new zealand, and chinese heart failure association. Eur J Heart Fail. (2021) 23:352–80. 10.1002/ejhf.2115 33605000

[103] Deng Y Wang J Xie G Zeng X Li H. Circ-HIPK3 strengthens the effects of adrenaline in heart failure by MiR-17-3p - ADCY6 axis. Int J Biol Sci. (2019) 15:2484–96. 10.7150/ijbs.36149 31595165 PMC6775314

[104] Baek SH. Beta blockers in heart failure: more evidence for an old friend. J Korean Med Sci. (2018) 33:e196. 10.3346/jkms.2018.33.e196 29915527 PMC6000598

[105] Morgan JP Erny RE Allen PD Grossman W Gwathmey JK. Abnormal intracellular calcium handling, a major cause of systolic and diastolic dysfunction in ventricular myocardium from patients with heart failure. Circulation. (1990) 81 (2 Suppl):III21–32. 2153479

[106] Wakili R Voigt N Kaab S Dobrev D Nattel S. Recent advances in the molecular pathophysiology of atrial fibrillation. J Clin Invest. (2011) 121:2955–68. 10.1172/JCI46315 21804195 PMC3148739

[107] Beazely MA Watts VJ. Regulatory properties of adenylate cyclases type 5 and 6: a progress report. Eur J Pharmacol. (2006) 535:1–12. 10.1016/j.ejphar.2006.01.054 16527269

[108] Shaywitz AJ Greenberg ME. CREB: a stimulus-induced transcription factor activated by a diverse array of extracellular signals. Annu Rev Biochem. (1999) 68:821–61. 10.1146/annurev.biochem.68.1.821 10872467

[109] Lv S Rong J Ren S Wu M Li M Zhu Y. Epidemiology and diagnosis of viral myocarditis. Hellenic J Cardiol. (2013) 54:382–91. 24100182

[110] Pollack A Kontorovich AR Fuster V Dec GW. Viral myocarditis–diagnosis, treatment options, current controversies. Nat Rev Cardiol. (2015) 12:670–80. 10.1038/nrcardio.2015.108 26194549

[111] Hou X Fu M Cheng B Kang Y Xie D. Galanthamine improves myocardial ischemia-reperfusion-induced cardiac dysfunction, endoplasmic reticulum stress-related apoptosis, and myocardial fibrosis by suppressing AMPK/Nrf2 pathway in rats. Ann Transl Med. (2019) 7:634. 10.21037/atm.2019.10.108 31930035 PMC6944599

[112] Fan S Hu K Zhang D Liu F. Interference of circRNA HIPK3 alleviates cardiac dysfunction in lipopolysaccharide-induced mice models and apoptosis in H9C2 cardiomyocytes. Ann Transl Med. (2020) 8:1147. 10.21037/atm-20-5306 33240996 PMC7576089

